# Thromboelastometry: Relation to the severity of liver cirrhosis in patients considered for liver transplantation

**DOI:** 10.1097/MD.0000000000007101

**Published:** 2017-06-08

**Authors:** Gabriel Dumitrescu, Anna Januszkiewicz, Anna Ågren, Maria Magnusson, Staffan Wahlin, Jan Wernerman

**Affiliations:** a Department of Anesthesia and Intensive Care Medicine, Karolinska University Hospital; b Department of Medicine, Division of Hematology, Coagulation Unit, Karolinska University Hospital; c CLINTEC, Division of Pediatrics, Astrid Lindgren Children's Hospital; d MMK, Clinical Chemistry and Blood Coagulation Research, Karolinska Institute; e Division of Hepatology, Centre for Digestive Diseases, Karolinska University Hospital, Stockholm, Sweden.

**Keywords:** Child-Pugh, hypercoagulability, liver failure, MELD, prognosis, thromboelastometry

## Abstract

The severity of liver disease is assessed by scoring systems, which include the conventional coagulation test prothrombin time-the international normalized ratio (PT-INR). However, PT-INR is not predictive of bleeding in liver disease and thromboelastometry (ROTEM) has been suggested to give a better overview of the coagulation system in these patients. It has now been suggested that coagulation as reflected by tromboelastomety may also be used for prognostic purposes. The objective of our study was to investigate whether thrombelastometry may discriminate the degree of liver insufficiency according to the scoring systems Child Pugh and Model for End-stage Liver Disease (MELD).

Forty patients with chronic liver disease of different etiologies and stages were included in this observational cross-sectional study. The severity of liver disease was evaluated using the Child-Pugh score and the MELD score, and blood samples for biochemistry, conventional coagulation tests, and ROTEM were collected at the time of the final assessment for liver transplantation. Statistical comparisons for the studied parameters with scores of severity were made using Spearman correlation test and receiver-operating characteristic (ROC) curves.

Spearman correlation coefficients indicated that the thromboelastometric parameters did not correlate with Child-Pugh or MELD scores. The ROC curves of the thromboelastometric parameters could not differentiate advanced stages from early stages of liver cirrhosis.

Standard ROTEM cannot discriminate the stage of chronic liver disease in patients with severe chronic liver disease.

## Abstract 2

Supplemental Digital Content is available in the text

## 1 Introduction

The underlying mechanisms affecting coagulation in advanced chronic liver disease have not been fully characterized. Decreased liver synthesis of coagulation factors and a lower platelet count are suggestive of bleeding diathesis. However, bleeding episodes are less frequent than may be expected based on conventional coagulation tests.^[1–3]^

The synthesis of anticoagulant proteins is also reduced in liver cirrhosis and thrombin generation is normal or even enhanced in patients with cirrhosis, if measured in the presence of thrombomodulin.^[4–6]^ In addition, an increased plasma concentration of factor VIII and von Willebrand factor as well as decreased levels of ADAMTS-13^[7]^ may compensate for thrombocytopenia.^[8,9]^ The modern concept of a re-balanced coagulation in liver cirrhosis states that “the average patient with liver failure may be in hemostatic balance despite prolonged routine coagulation tests.”^[10]^ An imbalance towards hypo- or hypercoagulation might still occur under certain clinical circumstances, especially in more advanced stages of liver insufficiency.^[11,12]^

Thromboelastography (TEG) and thromboelastometry (ROTEM) are global tests of coagulation that assess the viscoelastic properties of noncentrifuged blood that contains all blood cells and coagulation components. Clinically, these tests are mainly used to guide the administration of procoagulant treatment during and after surgical procedures that are associated with a high risk of massive bleeding, such as liver transplantation, trauma, or cardiac surgery. The use of TEG or ROTEM during liver transplantation may result in a significant reduction of blood and plasma transfusions by goal-directed procoagulant treatment.^[13,14]^ Furthermore, TEG and ROTEM are reported to enable detection of hypercoagulability and to predict thromboembolic events in patients undergoing major surgery.^[15–18]^

Tripodi et al^[19]^ has suggested that ROTEM, particularly maximum clot firmness (MCF), could be a good candidate to assess the severity of liver disease in patients with stable chronic liver disease. Today, 2 different scoring systems are used for the evaluation of severity of liver disease, the Child-Pugh score and the Model of End-Stage Liver Disease (MELD) score, which both include the conventional coagulation test prothrombin time-international normalized ratio (PT-INR).^[20–23]^

The primary aim of our study was to explore whether ROTEM has the potential to discriminate the degree of liver insufficiency according to the Child Pugh-score and the MELD score in unselected patients with liver disease considered for liver transplantation. In addition, the predictability of ROTEM for hypercoagulability, bleeding episodes, or massive bleeding during liver transplantation was observed.

## 2 Methods

### 2.1 Subjects

Patients with advanced liver disease (cirrhosis) referred for liver transplantation to the Division of Hepatology at Karolinska University Hospital Huddinge between December 2012 and March 2014 were included in the study. Coagulation disorders other than those related to liver disease as well as current medication that may affect coagulation (e.g., anticoagulation drugs or platelet aggregation inhibitors) were considered exclusion criteria. Patients with reversible complications such as current bacterial infections were also excluded. The patients gave written informed consent after being informed about the study protocol, both orally and in writing. The protocol was approved by the Regional Ethics Committee in Stockholm, Sweden, and was in accordance with the Helsinki Declaration of 1975.

### 2.2 Protocol

In conjunction with the final assessment for liver transplantation, blood samples for coagulation tests were collected through a peripheral venous catheter. At the same time, the severity of liver disease was evaluated using the Child-Pugh score and MELD score. All patients also underwent gastroscopy and imaging of the liver (ultrasound, computed tomography, or magnetic resonance tomography).

Gastrointestinal bleeding complications were registered retrospectively from 6 months before the time of sampling and prospectively until liver transplantation, or prospectively for 6 months in patients who did not undergo liver transplantation. Indirect signs of portal hypertension and portal vein thrombosis (PVT) were registered from imaging for all patients. Intraoperative bleeding and perioperative thrombosis complications during liver transplantation were also registered.

### 2.3 Analyses

Routine analyses were performed as follows: on a Sysmex CS 2100i (Sysmex Corporation, Kobe, Japan): PT-INR (with Owren method Stago SPA, Diagnostica Stago, Asnières sur Seine, France), activated partial thromboplastin time (with Stago PTT automate, Diagnostica Stago, Asnières sur Seine, France), fibrinogen (with the Clauss method, Siemens Thrombin reagent), antithrombin (AT) (with the anti-Xa based method, Innovance Siemens), d-dimers (with Roche Tinaquant reagents from Roche Diagnostics Ltd. Rotkreuz, Switzerland), platelet count, white blood cell count, and hemoglobin; on a BCS XP System (Siemens Healthcare Diagnostics Products GmbH, Marburg, Germany): protein C (with Berichrom PC, Siemens Healthcare Diagnostics Products GmbH) and protein S (with Coamatic PS-free, Chromogenix, Instrumentation Laboratory SpA Milano, Italy); on a Modular P EVO (Roche Diagnostics, Mannheim, Germany): total serum bilirubin (with BIL-T, Roche Diagnostics), serum albumin (with Albumin/BCP, Roche Diagnostics).

ROTEM was performed with a ROTEM delta device (Pentapharm GmbH, Munich, Germany). Coagulation was activated according to the instructions of the manufacturer (ellagic acid for INTEM, tissue factor for EXTEM, and tissue factor plus cytochalasin for FIBTEM). The analyzed thromboelastometric parameters for INTEM and EXTEM were: the clotting time (CT), representing the time in seconds from the start of the analysis to the recognizable initiation of clotting; clot formation time (CFT), representing the time in seconds from initiation of clotting until an amplitude of the graphical trace of 20 mm is established; and maximum clot firmness (MCF), representing the maximal amplitude (millimeters) of the graphical trace of clot firmness. For FIBTEM, only MCF was investigated.

The ROTEM assay was performed at the Department of Transfusion Medicine, whereas the other analyses were conducted at the Department of Clinical Chemistry.

### 2.4 Study size

Our study was designed to compare 2 groups of patients in different stages of liver cirrhosis according to the Child-Pugh score, specifically Child-Pugh A and B versus C.

The size of the samples was based on a power analysis (2-sided test) that assumed a difference of 1 standard deviation between the 2 groups, normal distribution, and alpha <0.05 for direct comparisons. A calculated minimum number of 17 patients in each group will then give a statistical power of 0.8. A sample of 19 from both positive and negative groups (allocation ratio 1:1) achieve a statistical power of 0.8 to detect a difference of 0.25 between the area under the ROC curve (AUC) under the null hypothesis of 0.50 and the AUC under the alternative hypothesis of 0.75 using a 2-sided *z* test at a significance level of *P* < .05; hence, we decided a study group size of 20 + 20 = 40 subjects. Power analysis was performed using PASS 2008 statistical software (NCSS, LLC, Kaysville, UT).

### 2.5 Statistics

Statistical comparisons for the studied parameters with scores of severity were made using Spearman correlation test. An *r* value >±0.3 was interpreted as an acceptable correlation and a *P* value ≤.05 was interpreted as statistical significant.

We generated ROC curves of MCF (INTEM, EXTEM, and FIBTEM) for 2 groups of patients classified according to Child-Pugh score. One group consisted of Child A and B patients (considered true negative), and the other group consisted of Child C patients (considered true positive). For comparison, using the same partition of the patients, we calculated ROC curves of serum bilirubin as well as of PT-INR and 1 procoagulant and 2 anticoagulant proteins synthesized by the liver (fibrinogen, AT, and protein C).

To evaluate the potential of ROTEM to assess survival, the patients were dichotomized also using a MELD score cut-off of 17, as mortality is reported to be higher above this value.^[24,25]^ We performed the same ROC curves as described above for these 2 groups of patients with MELD ≥17 (considered true positive) versus MELD <17 (considered true negative).

To evaluate the potential of ROTEM to predict bleeding risks, we generated ROC curves of CT and MCF (INTEM, EXTEM, and FIBTEM) for groups of patients dichotomized depending on bleeding episodes during the surviving period or on occurrence of massive bleeding during the liver transplantation (considered true positive). For comparison, using the same partition of the patients, we calculated ROC curves of routine coagulation tests. As the allocation ratio for these events deviated from 1:1, the statistical power associated with these calculations was given explicitly.

The frequency distribution of events such as bleeding in relation to different parameters was analyzed using Fisher exact test. No statistics were gathered on hypercoagulability owing to the low number of results in this area. The GraphPad Prism 5 statistical software package (GraphPad Software, Inc., San Diego, CA) was used for calculations.

Missing data were excluded from calculations.

## 3 Results

Forty patients with liver cirrhosis were enrolled in this study. Almost half the patients presented multiple etiologies. Patients’ characteristics and complications are presented in Table 1 and individual etiological diagnoses with existing associations between different etiologies are given in Table 2. The routine biochemical markers and coagulation tests as well the thromboelastometric parameters are presented in Table 3.

**Table 1:**
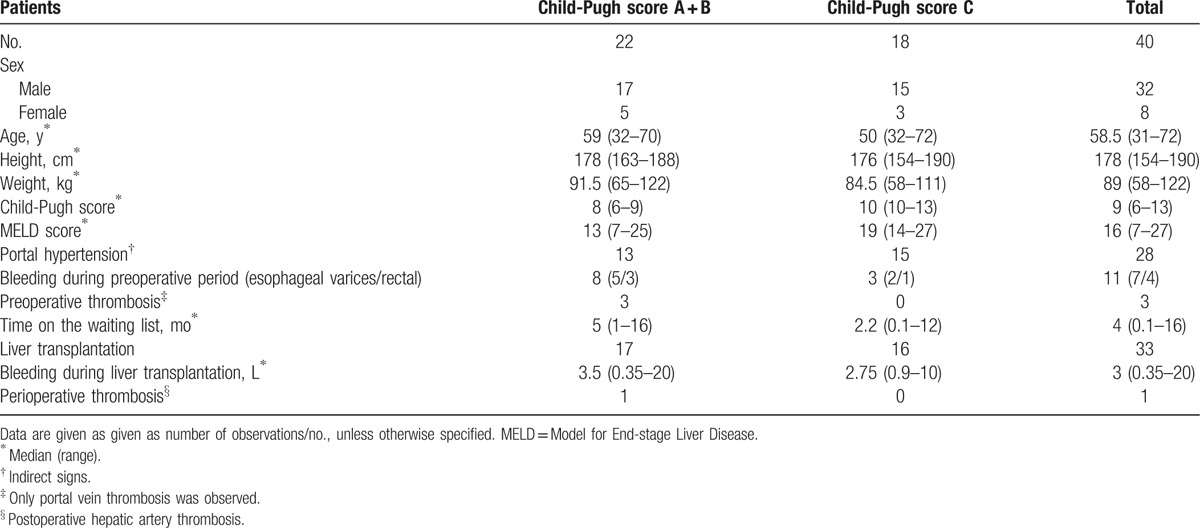
Patients’ characteristics, complications and surgical aspects.

**Table 2:**
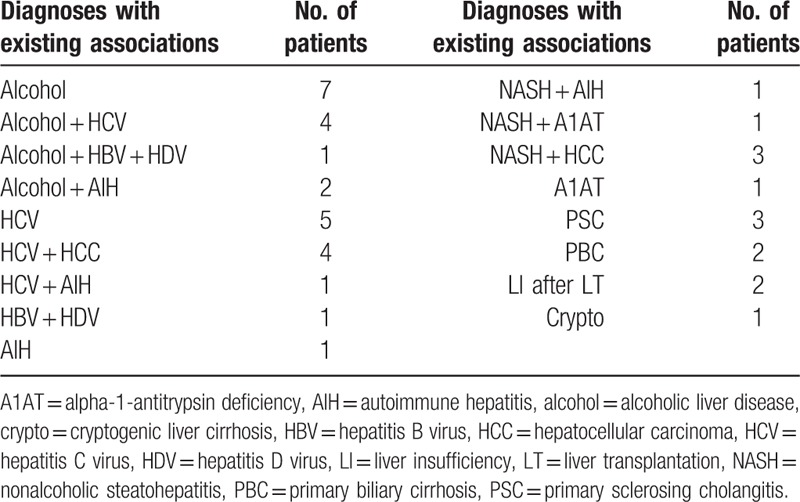
Patients’ individual diagnoses with existing associations between different etiologies in examined patients (n = 40) with liver failure.

**Table 3:**
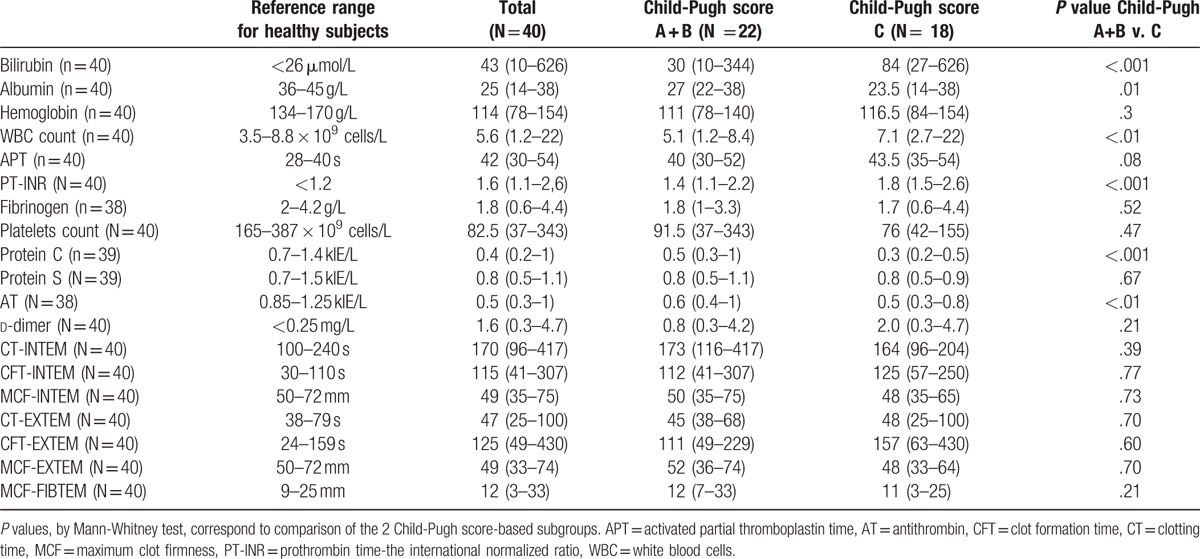
Patients’ biochemical and coagulation tests and thromboelastometric parameters, given as median (range).

**Figure 1:**
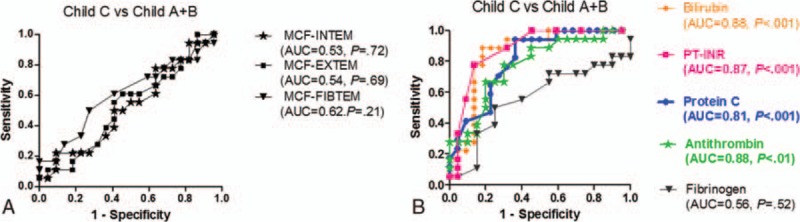
(A and B) Receiver-operating characteristic curves of different MCF analyses (A) and different biochemical and coagulation tests (B) in the patients (n = 18) with liver insufficiency in stage Child-Pugh C (considered true positive) versus patients (n = 22) in stage Child-Pugh A and B (considered true negative). The AUC and level of statistical significance (*P* value) are indicated in the parentheses for each parameter. AUC = area under the curve, MCF = maximum clot firmness.

See Figure 1, Supplemental Digital Content 1, which illustrates Spearman correlations between biochemistry, coagulation parameters parameters, and Child-Pugh score.

### 3.1 Thrombelastometry and child pugh score

**Figure 2:**
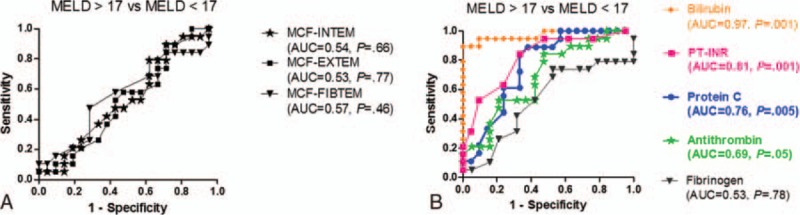
(A and B) Receiver-operating characteristic curves of different MCF-ROTEM analyses (A) and different biochemical and coagulation tests (B) in the patients (n = 19) with liver insufficiency in stage MELD over 17 (considered true positive) versus patients (n = 21) in stage MELD under 17 (considered true negative). The area under the curve (AUC) and level of statistical significance (*P* value) are indicated in the parentheses for each parameter. AUC = area under the curve, MELD = Model for End-stage Liver Disease, PT-INR = prothrombin time-the international normalized ratio.

Spearman correlation coefficients indicated that, with the exception of MCF-FIBTEM, the thromboelastometric parameters did not correlate with the Child-Pugh score. For all but 2 of the patients, thromboelastometric parameters were within the normal range or in the hypocoagulation area (see Figure 2, Supplemental Digital Content 2, which illustrates Spearman correlations between thromboelastometric parameters and Child-Pugh score). According to CT (both in INTEM and EXTEM), 95% of the patients included in our study were in the normal range. CFT and MCT did not indicate the same degree of coagulation balance; only 52% to 65% of CFT values (INTEM and EXTEM) and 45% of MCF values (INTEM and EXTEM) were in the normal range, whereas the rest indicated hypocoagulation.

ROC curves of MCF (in INTEM, EXTEM, and FIBTEM) for patients with Child-Pugh C (n = 18) versus patients with Child-Pugh A and B (n = 22) are presented in Figure 1A. The areas under the curve (AUC) for MCF (INTEM, EXTEM, and FIBTEM) did not reach statistical significance in differentiating between the 2 groups.

This is in contrast to ROC curves of bilirubin, PT-INR, protein C, and AT, which were highly statistically significant in differentiating between patients in stage Child-Pugh C and Child-Pugh A-B (Fig. 1, panel 1B).

### 3.2 Thrombelastometry and MELD score

The same poor correlation was observed between CT, CFT, and MCF and the MELD score (none of the Spearman correlation coefficients reached the value 0.3) (see Table, Supplemental Digital Content 3, which illustrates Spearman correlations between the thromboelastografic parameters and MELD score).

Figure 2 (panel 2A) shows the ROC curves of MCF (INTEM, EXTEM, and FIBTEM) for patients with MELD ≥17 (n = 19) versus patients with MELD <17 (n = 21). The ROC curves demonstrate, similar to the partition according to Child-Pugh score, that MCF failed to display the difference between the 2 MELD groups; this is in contrast to bilirubin, PT-INR, AT, and protein C, which proved to be able to differentiate the groups (Fig. 2, panel 2B).

### 3.3 Thrombelastometry and conventional coagulation factors

There were no significant correlations between the analyzed thromboelastometric parameters and bilirubin, activated partial thromboplastin time, PT-INR, or d-dimer. The parameter MCF (in INTEM, EXTEM, and FIBTEM) correlated well with fibrinogen (*r* = 0.7; *P* = .001) and platelet count (*r* = 0.7; *P* = .001) and, although to a lesser degree, with protein C (*r* = 0.4; *P* = .01) and AT (*r* = 0.4; *P* = .01) (Table 4). See Table, Supplemental Digital Content 4, which illustrates Spearman correlation coefficients between thromboelastometric parameters and all biochemical and routine coagulation tests.

**Table 4:**
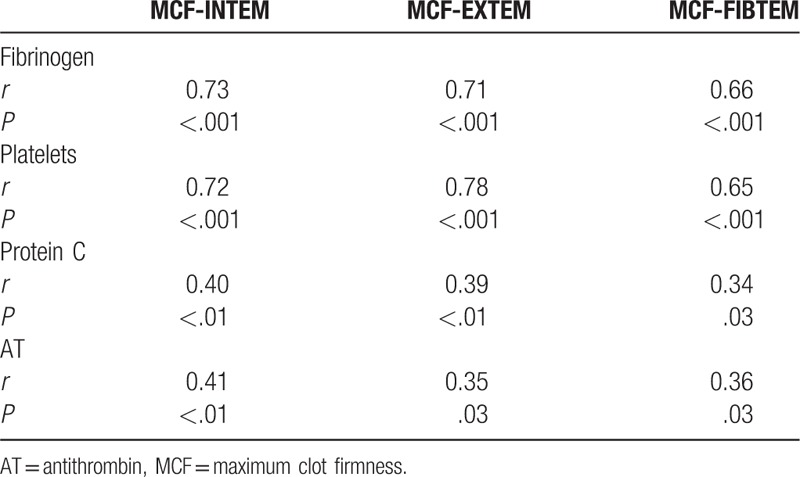
Spearman correlation coefficients *r* between MCF and fibrinogen, platelet count, protein C, and AT (n = 40).

### 3.4 Bleeding symptoms

**Figure 3:**
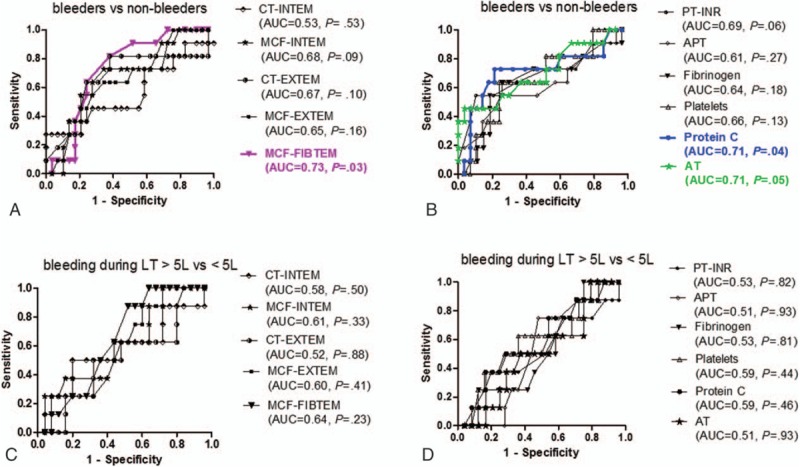
(A–D) Receiver-operating characteristic (ROC) curves of CT and MCF (ROTEM) and of routine coagulation tests in patients (n = 11) who bled during the surveillance period (considered true positive) versus those (n = 29) who did not bleed during the surveillance period (considered true negative). Panels 3 C-D: ROC curves of CT and MCF (ROTEM) and of routine coagulation tests in patients (n = 8) who had perioperative bleeding during liver transplantation of >5 L (considered true positive) versus those (n = 25) who bled <5 L (considered true negative). The area under the curve (AUC) and level of statistical significance (*P* value) are indicated in the parentheses for each parameter. APT = activated partial thromboplastin time, AT = antithrombin, AUC = area under the curve, CT = clotting time, MCF = maximum clot firmness, PT-INR = prothrombin time-the international normalized ratio.

Eleven patients presented bleeding episodes during the prospective observation period while waiting for liver transplantation. Seven patients experienced bleeding from esophageal varices, and 4 patients had rectal bleedings from hemorrhoids. Figure 3A and B present the ROC curves of the thromboelastometric parameters CT and MCF as well of routine coagulation tests for the patients (n = 11) who bled versus those who did not. A statistically significant difference between the 2 groups was only found for protein C, AT, and MCF-FIBTEM (statistical power for an AUC of 0.75 was 70%). When explored in detail with Fisher exact test, the bleeding episodes were rare in patients with a low MCF-FIBTEM (cutoff value 11 mm), compared to patients with higher values (*P* = .03). See Table E and F, Supplemental Digital Content 5, which illustrates the numeric values of the AUC, the 95% confidence interval, and the level of statistical significance for the ROC curves.

No significance in association was found between the bleeding episodes and the presence of indirect signs of portal hypertension.

Among the 40 patients included in the study, 6 were not accepted for liver transplantation (2 were considered to be in a too-early disease stage for liver transplantation and 4 had contraindications) and 1 additional patient died while on the waiting list. Finally, 33 patients underwent liver transplantation. Eight of these patients had massive intraoperative bleeding (defined as >5 L). Figure 3 C and D show the ROC curves of the ROTEM parameters CT and MCF as well of routine coagulation tests for the patients (n = 8) with massive intraoperative blood loss versus the others. AUCs of these parameters did not reach statistical significance in differentiating between the 2 groups (statistical power for an AUC of 0.75 was 57%). See Table G and H, Supplemental Digital Content 5, which illustrates the numeric values of the AUC, the 95% confidence interval, and the level of statistical significance for the ROC curves.

### 3.5 Thrombotic symptoms

Three of 40 patients (i.e., 7.5% of the patients included in the study) were diagnosed with PVT but had no signs of hypercoagulability on ROTEM. Two of 40 patients without signs of portal venous thrombosis presented signs of hypercoagulability on ROTEM. One of these patients had a history of pulmonary embolism before the study protocol and eventually developed hepatic artery thrombosis after the liver transplantation.

## 4 Discussion

The results of our study suggest that standard ROTEM does not have associations with the staging of liver disease assessed by conventional risk scores in patients with chronic liver disease of mixed etiologies evaluated for liver transplantation. In this study of pilot character, no attempt was made to directly link ROTEM to outcomes, something which would have demanded a sample size of a different magnitude. However, the inability for ROTEM to discriminate between liver insufficiency of moderate and severe degree discourages such an effort.

We found that the ROTEM did not statistically correlate with the Child-Pugh and MELD scores and could not differentiate between early and advanced stages of liver disease. We were not able to demonstrate that MCF differentiates stage Child-Pugh A/B from Child-Pugh C or to discern the groups dichotomized according to MELD score with a cut-off of 17. In earlier studies, wherein MCF is reported to correlate with the severity of liver disease, the case-mix of the cohorts studied has been quite different, with a minority of patients in the group of Child-Pugh C/MELD >16.^[19,26]^ Furthermore, correlations and ROC curves reflect different aspects.

PT-INR values according to the Owren method depend on the levels of the coagulation factor II (FII), FVII, and FX, all synthesized in the liver.^[27]^ An impaired synthetic function of the hepatocytes in liver failure results in general in reduced levels of these coagulant factors, which lead to an increased PT-INR. This is the rationale behind the use of PT-INR in severity scores for patients with liver failure. The Child-Pugh score is also dependent on serum bilirubin, serum albumin, ascites, and encephalopathy and the MELD score includes serum bilirubin and serum creatinine apart from PT-INR.^[21,28]^ On the contrary, ROTEM is a global coagulation test, which depends on multiple parameters with impact on hemostasis including both pro- and anticoagulation factors. According to the theory of rebalanced coagulation in liver failure, the sum of the reductions in both pro- and anticoagulant factors leads to a new balance in the coagulation. Although fragile, this new balance may provide a more normal thromboelastometric curve than would be expected if only the PT-INR should be considered. In our study, the thromboelastometric results were within the normocoagulable or hypocoagulable range to the same extent in patients with mild-moderate as in severe liver failure. This may indicate a type of rebalanced coagulation irrespective of the stage of liver failure and would explain the lack of correlation with the severity scores. This is in contrast with 2 previous studies regarding patients with a higher degree of etiological homogeneity based on alcoholic and viral liver disease, which showed a degree of correlation between MCF and Child-Pugh and MELD score, respectively.^[19,26]^ It can be noted that PT-INR according to Quick was used in their scoring systems. The Quick method is dependent on fibrinogen and FV in addition to FII, FVII, and FX.^[27]^

The absence of a significant correlation between MCF and the severity of liver disease could also result from the influences that fibrinogen and platelets exert on this thromboelastometric parameter. Indeed, in our patients, neither fibrinogen nor platelets correlated with the severity scores, and it has been demonstrated that MCF is essentially altered by these 2 factors.^[29]^

We observed a definitively negative correlation between the Child-Pugh score and AT and protein C, which was perhaps not surprising, as the hepatic synthesis of proteins decreases in advanced stages of disease. Furthermore, protein C and AT could differentiate Child-Pugh A-B from Child-Pugh C and could differentiate between MELD scores below or over 17. The potential value of protein C and AT in improving accuracy when estimating the degree of liver insufficiency remains to be established.^[30]^

Our study confirms the earlier reported discrepancy between the thromboelastometric parameter CT and PT-INR in patients with liver cirrhosis.^[19]^ This contrasts with what is observed in non-cirrhotic patients treated with warfarin, wherein a good correlation between CT-EXTEM and PT-INR is reported.^[31]^

Our results are in accord with earlier reports that ROTEM does not indicate hypercoagulability in chronic liver disease.^[26,32]^ In particular, the patients with Child-Pugh C did not have an explicit hypercoagulability as has been suggested by studies using thrombin generation measured with thrombomodulin.^[5,12]^ Expression of hypercoagulability on TEG varies among liver diseases classically considered as potential hypercoagulable. Primary biliary cirrhosis and primary sclerosing cholangitis may present to a considerable degree thromboelastographic signs of hyperactive coagulation,^[16,33]^ whereas hepatocellular carcinoma in the absence of cirrhosis does not.^[34]^ Because the sensitivity differs between TEG and ROTEM,^[29]^ the results from one assay cannot be extrapolated to the other.

We also noted episodes of bleeding and thrombosis, as well as massive bleeding during liver transplant surgery. This part of the study was only hypothesis-generating as the material was insufficient for any conclusions. The only ROTEM parameter studied that could possibly predict bleeding episodes during the observational period was MCF-FIBTEM, which could separate bleeders from nonbleeders. Surprisingly, we found that it was the individuals with low MCF-FIBTEM who did not bleed, suggesting that a low value was not associated with an increased risk of bleeding. Interestingly, plasma fibrinogen concentration did not separate bleeders from nonbleeders. Still, MCF-FIBTEM showed that 80% of the patients were within the normal range, although a majority had lower than normal plasma fibrinogen concentrations (57.5% of the plasma fibrinogen values were below the normal range).

Another interesting finding was that protein C and AT plasma levels could separate bleeders from nonbleeders on the waiting list; lower plasma levels were associated with less bleeding events.

The failure of thromboelastometric parameters, measured at the time of pretransplant evaluation, to predict massive intraoperative bleedings during liver transplantation is less surprising. Intraoperative bleeding is probably related mainly to surgical factors rather than to coagulation status.^[2]^

The strengths of our study are that we studied a patient group with heterogeneous etiologies of liver disease typical for an European liver transplantation center,^[35]^ thus increasing the external validity; we studied a well-balanced distribution of Child-Pugh and MELD scores respectively, comparable to what is internationally reported for patients assigned for liver transplantation^[36]^; and both pro- and anticoagulant mechanisms were studied.

Our study also has some limitations: clotting factors and biomarkers for thrombin generation and fibrinolysis (except for d-dimers) were not included; owing to the limited size of this pilot study, the numbers of bleeding and thrombosis complications were too low to allow conclusions; and we did not systematically screen for subclinical deep vein thrombosis.

## 5 Conclusions

ROTEM assessed in cirrhotic patients of mixed etiologies demonstrated almost exclusively a normocoagulable or hypocoagulable state, regardless of the severity of the chronic liver disease. There was no correlation between the standard thromboelastometric parameters and the severity of liver disease. Based on our results, ROTEM seems to have no role in the clinical evaluation of the severity of chronic liver disease in unselected patient groups.

## Acknowledgments

The authors gratefully acknowledge the skilled nursing assistance of Ms. Kristina Kilsand, Ms. Lena Nyström, and Ms. Sara Rydén, as well as the skilled technical assistance of Ms. Gunilla Gryfelt. The authors wish to acknowledge Dr. Åke Norberg for the priceless statistical advices given.

## Supplementary Material

Supplemental Digital Content
