# Thromboelastometry: Relation to the severity of liver cirrhosis in patients considered for liver transplantation: Erratum

**DOI:** 10.1097/MD.0000000000007420

**Published:** 2017-06-30

In the article, “Thromboelastometry: Relation to the severity of liver cirrhosis in patients considered for liver transplantation”,^[1]^ which appeared in Volume 96, Issue 23 of *Medicine*, “Thrombelastometry and child pugh score” should have appeared as “Thrombelastometry and Child-Pugh score.”
