# Supplementary material for: Thromboelastometry: Relation to the severity of liver cirrhosis in patients considered for liver transplantation
Source: Medicine (Baltimore). 2017 Jun 8;96(23):e7101. doi: 10.1097/MD.0000000000007101 (PMC5466232; doi:10.1097/MD.0000000000007101)
Supplement: Supplemental Digital Content [file medi-96-e7101-s001.pdf]

**Figure 1 (A-J).** Biochemistry and coagulation parameters in patients with liver insufficiency (n=40). The normal range is indicated by the shaded area. A vertical line separates the patients into Child-Pugh A/B or Child-Pugh C stage groups. Spearman's correlation coefficients (r value) and the levels of statistical significance (*P* value) are indicated in the right upper corner.

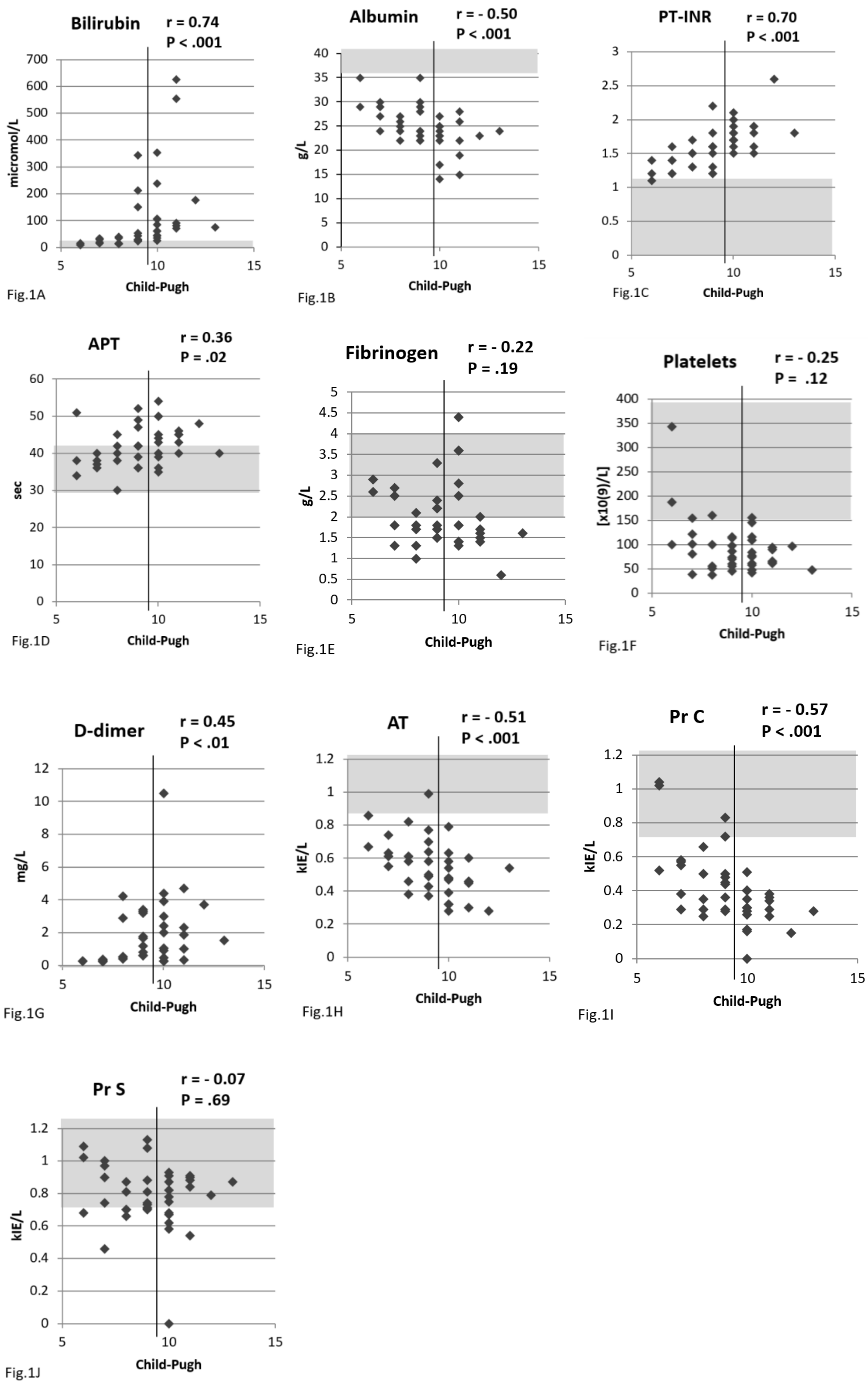

**Figure 2 (A-G)**. Thromboelastometric parameters in patients with liver insufficiency (n=40). The normal range is indicated by the shaded area. A vertical line separates the patients into Child-Pugh A/B or Child-Pugh C stage groups. Spearman's correlation coefficients (r value) and the levels of statistical significance (p value) are indicated in the right upper corner.

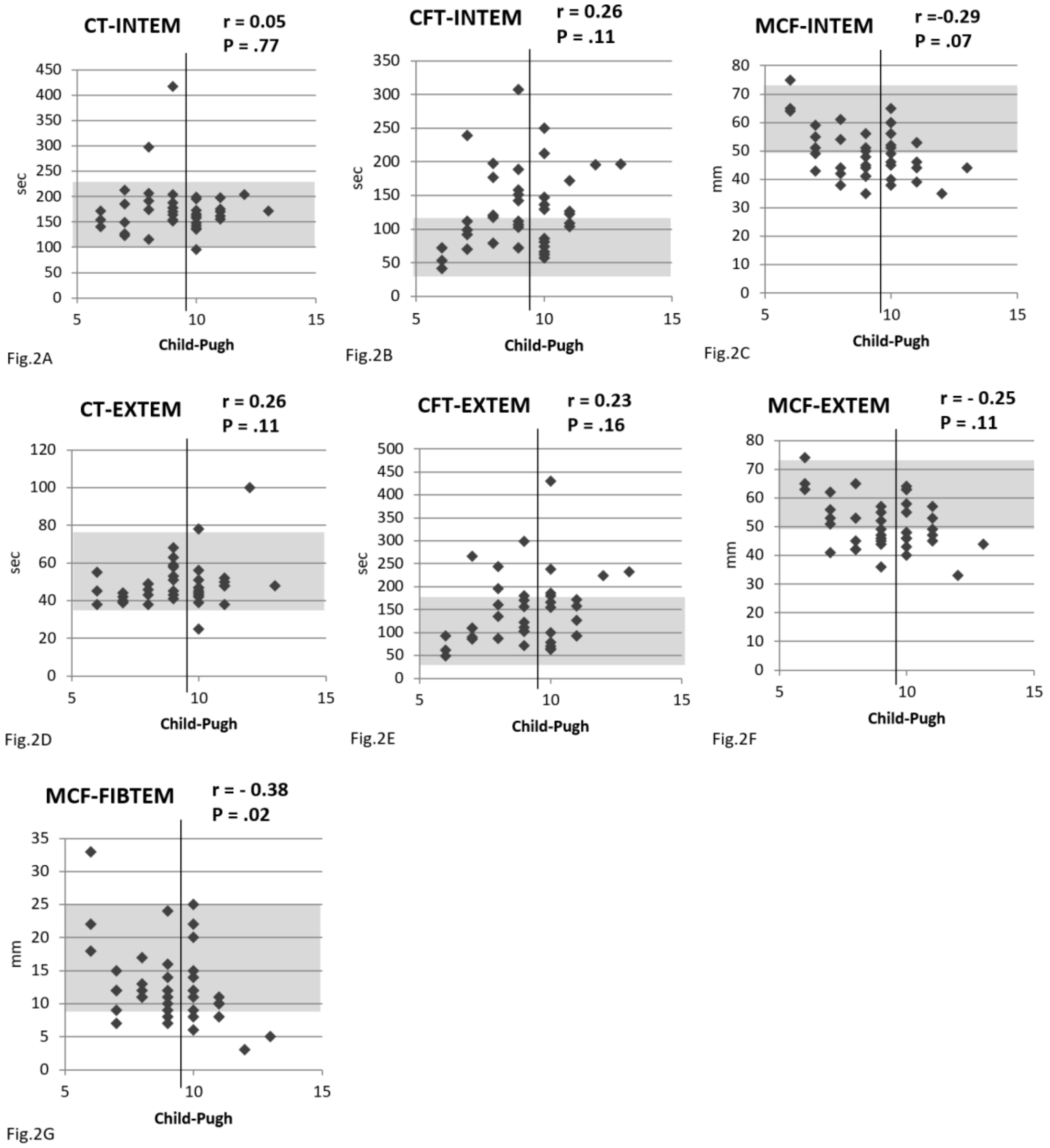

**Table**. Spearman `s correlations between the thromboelastografic parameters and MELD score (n=40).

| | | CT INTEM | CFT INTEM | MCF INTEM | CT EXTEM | CFT EXTEM | MCF EXTEM | MCF FIBTEM |
|---|---|---|---|---|---|---|---|---|
| MELD score | **Spearman r** | **0.20** | **0.14** | **-0.24** | **0.27** | **0.12** | **-0.21** | **-0.29** |
| | *P* value | .21 | .37 | .13 | .09 | .47 | .19 | .07 |

**Table**. Spearman's correlation coefficients r between thromboelastometric parameters and biochemical and routine coagulation tests (n=40).

| | CT-INTEM | CFT-INTEM | MCF-INTEM | CT-EXTEM | CFT-EXTEM | MCF-EXTEM | MCF-FIBTEM |
|---|---|---|---|---|---|---|---|
| Bilirubin | 0.15 | 0.06 | -0.21 | 0.34 | 0.03 | -0.16 | -0.20 |
| APT | 0.07 | 0.04 | -0.14 | 0.11 | -0.04 | -0.07 | -0.22 |
| PT-INR | 0.19 | 0.24 | -0.30 | 0.27 | 0.18 | -0.28 | -0.30 |
| Fibrinogen | -0.22 | -0.78 | 0.73 | -0.27 | -0.63 | 0.71 | 0.66 |
| Protein C | -0.20 | -0.37 | 0.40 | -0.11 | -0.34 | 0.39 | 0.34 |
| AT | -0.13 | -0.32 | 0.41 | -0.20 | -0.23 | 0.35 | 0.36 |
| Protein S | -0.04 | -0.49 | 0.26 | -0.09 | -0.40 | 0.32 | 0.32 |
| Platelets | -0.41 | -0.83 | 0.72 | -0.26 | -0.80 | 0.78 | 0.65 |
| D-dimer | -0.21 | 0.19 | -0.22 | -0.03 | 0.15 | -0.20 | -0.22 |

**Table A-H**

**Table A.** The numeric values of the areas under the curve (AUC) , the 95% confidence interval and the level of statistic significance for the ROC curves of biochemistry, conventional coagulation tests for patients (n=18) with liver insufficiency in stage Child-Pugh C (considered true positive) versus patients (n=22) in stage Child-Pugh A and B (considered true negative)

| | AUC | 95% confidence interval | *P* value |
|---|---|---|---|
| Bilirubin | 0.88 | 0.77 to 0.99 | <.001 |
| Albumin | 0.73 | 0.58 to 0.89 | .01 |
| PT-INR | 0.88 | 0.77 to 0.99 | <.001 |
| Fibrinogen | 0.56 | 0.37 to 0.75 | .52 |
| Protein C | 0.81 | 0.67 to 0.94 | <.001 |
| Antithrombin | 0.77 | 0.62 to 0.92 | <.01 |

**Table B.** The numeric values of the areas under the curve (AUC) , the 95% confidence interval and the level of statistic significance for the ROC curves of thromboelastometric parameters for patients (n=18) with liver insufficiency in stage Child-Pugh C (considered true positive) versus patients (n=22) in stage Child-Pugh A and B (considered true negative)

| | AUC | 95% confidence interval | *P* value |
|---|---|---|---|
| CT-INTEM | 0.58 | 0.40 to 0.76 | .39 |
| CFT-INTEM | 0.53 | 0.35 to 0.71 | .76 |
| MCF-INTEM | 0.53 | 0.35 to 0.71 | .72 |
| CT-EXTEM | 0.54 | 0.35 to 0.72 | .69 |
| CFT-EXTEM | 0.55 | 0.36 to 0.73 | .60 |
| MCF-EXTEM | 0.54 | 0.35 to 0.72 | .69 |
| MCF-FIBTEM | 0.62 | 0.44 to 0.79 | .21 |

**Table C.** The numeric values of the areas under the curve (AUC) , the 95% confidence interval and the level of statistic significance for the ROC curves of biochemistry, conventional coagulation tests for patients (n=19) with liver insufficiency in stage MELD over 17 (considered true positive) versus patients (n=21) in stage MELD under 17 (considered true negative)

| | Area | 95% confidence interval | *P* value |
|---|---|---|---|
| Bilirubin | 0.97 | 0.92 to 1.02 | <.001 |
| Albumin | 0.64 | 0.46 to 0.81 | .13 |
| PT-INR | 0.82 | 0.68 to 0.95 | <.001 |
| Fibrinogen | 0.53 | 0.34 to 0.72 | .78 |
| Protein C | 0.76 | 0.61 to 0.91 | <.01 |
| Antithrombin | 0.69 | 0.52 to 0.86 | .05 |

**Table D.**The numeric values of the areas under the curve (AUC) , the 95% confidence interval and the level of statistic significance for the ROC curves of thromboelastometric parameters for patients (n=19) with liver insufficiency in stage MELD over 17 (considered true positive) versus patients (n=21) in stage MELD under 17 (considered true negative)

| | Area | 95% confidence interval | *P* value |
|---|---|---|---|
| CT-INTEM | 0.57 | 0.39 to 0.75 | .44 |
| CFT-INTEM | 0.50 | 0.32 to 0.68 | .99 |
| MCF-INTEM | 0.54 | 0.36 to 0.72 | .66 |
| CT-EXTEM | 0.60 | 0.42 to 0.78 | .28 |
| CFT-EXTEM | 0.50 | 0.32 to 0.68 | .98 |
| MCF-EXTEM | 0.53 | 0.35 to 0.71 | .77 |
| MCF-FIBTEM | 0.57 | 0.37 to 0.75 | .46 |

**Table E.** The numeric values of the areas under the curve (AUC) , the 95% confidence interval and the level of statistic significance for the ROC curves of conventional coagulation tests for patients (n=11) who bled during the surveillance period (considered true positive) versus those (n= 29) who did not bleed during the surveillance period (considered true negative)

| | Area | 95% confidence interval | *P* value |
|---|---|---|---|
| PT-INR | 0.69 | 0.48 to 0.90 | .06 |
| APT | 0.61 | 0.40 to 0.81 | .27 |
| Fibrinogen | 0.64 | 0.43 to 0.85 | .18 |
| Platelets | 0.66 | 0.47 to 0.84 | .13 |
| Protein C | 0.71 | 0.50 to 0.92 | .04 |
| Antithrombin | 0.71 | 0.51 to 0.90 | .05 |

**Table F.**The numeric values of the areas under the curve (AUC) , the 95% confidence interval and the level of statistic significance for the ROC curves of thromboelastometric parameters for patients (n=11) who bled during the surveillance period (considered true positive) versus those (n= 29) who did not bleed during the surveillance period (considered true negative)

| | Area | 95% confidence interval | *P* value |
|---|---|---|---|
| CT-INTEM | 0.56 | 0.34 to 0.79 | .53 |
| CFT-INTEM | 0.63 | 0.45 to 0.81 | .20 |
| MCF-INTEM | 0.68 | 0.49 to 0.86 | .09 |
| CT-EXTEM | 0.67 | 0.46 to 0.88 | .10 |
| CFT-EXTEM | 0.63 | 0.46 to 0.81 | .19 |
| MCF-EXTEM | 0.65 | 0.46 to 0.83 | .16 |
| MCF-FIBTEM | 0.73 | 0.57 to 0.89 | .03 |

**Table G.** The numeric values of the areas under the curve (AUC) , the 95% confidence interval and the level of statistic significance for the ROC curves of conventional coagulation tests for patients (n=8) who had perioperative bleeding during liver transplantation of more than 5 L (considered true positive) versus those (n= 25) who bled less than 5 L (considered true negative).

| | Area | 95% confidence interval | *P* value |
|---|---|---|---|
| PT-INR | 0.53 | 0.28 to 0.77 | .82 |
| APT | 0.51 | 0.30 to 0.72 | .93 |
| Fibrinogen | 0.53 | 0.31 to 0.75 | .81 |
| Platelets | 0.59 | 0.36 to 0.82 | .44 |
| Protein C | 0.59 | 0.37 to 0.81 | .46 |
| Antithrombin | 0.51 | 0.29 to 0.73 | .93 |

**Table H.**The numeric values of the areas under the curve (AUC) , the 95% confidence interval and the level of statistic significance for the ROC curves of thromboelastometric parameters for patients (n=8) who had perioperative bleeding during liver transplantation of more than 5 L (considered true positive) versus those (n= 25) who bled less than 5 L (considered true negative).

| | Area | 95% confidence interval | *P* value |
|---|---|---|---|
| CT-INTEM | 0.58 | 0.33 to 0.83 | .50 |
| CFT-INTEM | 0.63 | 0.42 to 0.84 | .27 |
| MCF-INTEM | 0.61 | 0.39 to 0.83 | .33 |
| CT-EXTEM | 0.52 | 0.29 to 0.75 | .88 |
| CFT-EXTEM | 0.63 | 0.42 to 0.84 | .27 |
| MCF-EXTEM | 0.60 | 0.39 to 0.80 | .41 |
| MCF-FIBTEM | 0.64 | 0.45 to 0.83 | .23 |
